# Correlation between coronary artery calcification by non-cardiac CT and Framingham score in young patients

**DOI:** 10.1371/journal.pone.0195061

**Published:** 2018-03-28

**Authors:** Gabriel Lichtenstein, Amichai Perlman, Shoshana Shpitzen, Ronen Durst, Dorit Shaham, Eran Leitersdorf, Auryan Szalat

**Affiliations:** 1 Center for Research, Prevention and Treatment of Atherosclerosis, Hadassah-Hebrew University Medical Center, Jerusalem, Israel; 2 Internal Medicine Ward, Hadassah-Hebrew University Medical Center, Jerusalem, Israel; 3 Heart Institute, Hadassah-Hebrew University Medical Center, Jerusalem, Israel; 4 Medical Imaging Department, Hadassah-Hebrew University Medical Center, Jerusalem, Israel; University of Colorado Denver School of Medicine, UNITED STATES

## Abstract

**Background:**

Previous studies have established a correlation between coronary artery calcification (CAC) measured by ECG-gated chest computed tomography (CT) and cardiovascular disease. Recent reports which included asymptomatic patients suggest that CAC measured on non-ECG gated CT is similarly associated with cardiovascular risk. This study investigates the correlation between the Framingham Risk Score (FRS) and an incidental finding of CAC on a non-gated chest CT performed for non-cardiac indications in young and seemingly healthy adults.

**Methods:**

A cross-sectional study that included 162 CT scans performed in young patients aged 18–50 years old for non-cardiac indications in our institution was conducted. CAC score (CACS) was calculated using the Agatston method. FRS was calculated and compared to the CACS using three different approaches. The correlations between the CACS and several specific factors (i.e. age, body mass index, smoking, statins, etc.), were also evaluated.

**Results:**

Mean age of patients was 36.43 year old and 105 (64.8%) were male. We found a significant positive correlation between the CACS and the FRS in all three approaches (p<0.05). Increased age, smoking and statin use were the only individual factors clearly associated with an increase in CACS (p = 0.002, p = 0.045 and p = 0.009, respectively).

**Conclusion:**

This is the first report indicating that incidental CACS identified in non-gated MDCT is also associated with cardiovascular risk evaluated by FRS in a young population. Our findings suggest that young asymptomatic individuals with incidental CAC should be seriously evaluated for cardiovascular risk factors despite presumption of belonging to a low cardiovascular risk category.

## Introduction

Coronary artery disease (CAD) is a major cause of morbidity and mortality in developed countries. Despite a reduction in mortality rates in recent decades, CAD is still responsible for about one third of all deaths among individuals over 35 years of age in the developed world [1]. Great efforts are invested in primary prevention and diagnosis of asymptomatic disease by identification and control of known and established cardiovascular risk factors (CVRF) (i.e. age, sex, hypertension, hyperlipidemia, smoking, family history, obesity, and diabetes). Several cardiovascular risk scores based on large epidemiological studies and employing combinations of these independent risk factors are available. One of the best known and most commonly used of these scores is the Framingham Risk Score (FRS) derived from the Framingham Heart Study [2]. However, existing scores still have only moderate precision, and additional factors and markers are being explored to help better estimate an individual’s cardiovascular risk [3]. A few studies have established the correlation between coronary artery calcification score (CACS), measured using Electron Beam Computed Tomography (EBCT) with the Agatston method [4], and coronary artery disease in an asymptomatic population [5–9]. Thus, CACS has been described as a new cardiovascular prognostic risk factor [10–15] with better accuracy than other independent CV risk factors, such as the C-Reactive Protein (CRP), and it has been suggested it can serve to improve the FRS [9].

In order to achieve a reliable CAC measurement, methods to reduce imaging artifacts, caused by the motion of the respiratory system and heart, have been developed. The EBCT machines were the first to enable rapid image acquisition during breath-holding. However, in the last couple of decades Multidetector CT (MDCT) technology [16] showed similar accuracy. Newer and more technologically advanced MDCT machines allowed a rapid enough image acquisition to achieve a diagnostic image even without ECG gating. A few studies [17–19] have reported good correlation between the measurement of coronary calcification using ECG-gated MDCT and non-gated MDCT. In some studies, CAC measurement by non-gated MDCT revealed prognostic information regarding the risk for cardiovascular event and mortality [20, 21].

As chest CT is today a frequently used imaging test, CAC are more frequently observed and should be more reported [22]. Indeed, correlation to cardiovascular prognosis and mortality exists, mostly in patients older than 40 year old [23].

However, it is important to understand whether the observation of CAC in young patients is indicative of cardiovascular risk, and whether it may require further investigation. To our knowledge, the association between CAC and early CV risk factors in young individuals up to 45 year old has been shown in the CARDIA study [24] and in the Dallas Heart Study [25] using cardiac gated-CT or EBM-CT but no data is available in regard to CAC observed in this population on non-cardiac CT.

The aim of our study was to evaluate the correlation between CAC, as an incidental finding detected by routine non-gated CT, and cardiovascular risk in young adults with no previously documented cardiovascular diseases.

## Methods

### Population and setting

We scanned the Hadassah Hebrew University Medical Center databases to identify individuals aged 18 to 50 years, who underwent non-gated chest MDCT for non-cardiac indication between July 2005 and May 2013. We reviewed the patients’ medical history via the hospital’s medical management program system and excluded subjects who were diagnosed prior to the scan with ischemic heart disease, malignancy, chronic inflammatory disease, calcium disorder, impaired renal function (glomerular filtration rate < 60 ml/ minute), and subjects who received bisphosphonates or steroids. The study was approved by the Hadassah-Hebrew University Hospital ethics committee for clinical research. The latter waived the requirement for patients' informed consent.

### Measurements and co-variates

During this period, chest-CT was performed using a thirty-two-detector row CT. Every patient’s CACS was calculated using the Agatston method with a dedicated software (HeartBeat–CS in a Philips EBW workstation v4.5). For each patient we also calculated the Framingham Risk score (FRS) based on the patients' recorded age, sex, total and high-density lipoprotein (HDL) cholesterol levels, smoking status, and systolic blood pressure. In addition to the described risk factors, we also documented additional factors in order to determine their possible link to coronary calcification, which included: body mass index (BMI), 25-hydroxy-vitamin D level, diabetes mellitus (DM) status and the use of statins and anti-hypertensive medications.

### Data analysis

The correlation between CACS and FRS was evaluated using three approaches. In the first approach CACS was dichotomized, and coded as present for participants with any calcification (CACS>0), and absent for participants with no calcification (CACS = 0), while FRS was divided into three categories: (1) "Low" (FRS<1%), (2) "Medium" (1%≤FRS≤5%), and (3) "High" (FRS>5%). In the second approach, CACS was categorized as “Low” (CACS = 0), “Medium” (CACS between 0–10), or “High” (CACS≥10), while FRS was again divided into three categories. In the third approach, FRS was evaluated as a continuous variable, while CACS was dichotomized. In addition to the association between CAC and Framingham Score, we evaluated the univariate association between specific FRS components and coronary calcification. We also examined the univariate association between CAC and a number of additional laboratory and health related measures, including the presence of diabetes, use of anti-hypertensive medication, and use of statins.

### Statistics

Descriptive statistics are presented using mean and standard deviation (SD) for normally distributed quantitative variables, and the median and interquartile range (IQR) for non-normally distributed variables. Associations between variables were evaluated using appropriate statistical methods (Chi-square or Fisher's exact test were used to test for differences in categorical variables, Cramer’s V to quantify correlation between categorical variables, linear trends for ordinal variables were tested using Mantel-Haenszel’s test for linear trend, t-tests or comparable non-parametric methods were used to evaluate differences in continuous variables). All statistical tests were bidirectional, and a p-value smaller or equal to 5% was considered statistically significant. In order to test the possibility of an inter-observer variability, 23 scans were reanalyzed using the designated software by an imaging expert. McNemar-Bowker test was performed and Cohens κ statistic for the proportion of agreement was tested. The inter-observer concordance was assessed for four score ranks (0, 1–10, 10–100, >100). A Cohen’s κ statistic of less than 0.2 indicated poor agreement, 0.21–0.4 fair agreement, 0.41–0.6 moderate agreement, 0.61–0.8 good agreement and 0.81–1 excellent agreement. To rule out intra-observer variability, the first 30 scans analyzed were reanalyzed for a second time at the end of the study scan analysis. Statistical analyses were performed using SAS software.

## Results

### Study population

We identified 1154 CT scans performed in young adults between the ages 18 to 50 during the prescribed period. We excluded 992 subjects based on exclusion criteria or non-readable CT, leaving 162 subjects for whom both CACS evaluation and FRS calculation were obtained for analysis. All the characteristics of the participants included in the study are detailed in Table 1. Data regarding BMI was available for 69 (42.6%) of the subjects, and serum 25-hydroxy-vitamin-D levels for 60 (37%).

**Table 1:** Characteristic of study participants.

	n = 162
Age, mean (SD)	36.43 (9.4)
Sex (Females/Males)	57/105
History of smoking, n (%)	59 (36.4%)
Total cholesterol (mg/dL), mean (SD)	181.6 (37.1) [4.7 mmol/L (0.96)]
HDL (mg/dL), mean (SD)	44.9 (12.6) [1.16 mmol/L (0.32)]
Systolic blood pressure (mmHg), mean (SD)	125 (15.6)
BMI (Kg/m^2^), median (IQR)	24.9 (7.2)
25-hydroxy vitamin D level (nmol/L) (normal range 20–100), median (IQR)	17.5 (12.375)

HDL, High-density lipoprotein; BMI, Body mass index.

The patients included in the analysis were generally under 40 years of age (mean age 36.43), and mostly male (64.8%). A history of smoking was observed in 59 patients (36.4%). Six patients were previously diagnosed with type 2 DM (3.7%), five (3.1%) were treated using anti-hypertensive medications and 7 (4.3%) using statins. When calculating the FRS, we found that 69 (42.6%) of the subjects had a FRS lower than 1%, while 33 (20.4%) had a FRS higher than 5%.

Indications for MDCT were: respiratory symptoms (upper respiratory tract infections, pneumonia, cough and shortness of breath) in 88 patients (54.3%), evaluation of an abnormal finding on chest radiography or follow-up of lung nodules in 33 patients (20.3%), trauma in 16 patients (9.9%), spontaneous pneumothorax in 13 patients (8.1%), screening for lung cancer in 11 patients (6.8%), pre-operative evaluation in 1 patient (0.6%). Evidence of any coronary calcification was detected in 19 (11.7%) subjects, and seven of the participants (4.3%) had a CACS>10. Participants with evidence of coronary calcification were on average older, more likely to be male, and more likely to have smoked.

### Coronary artery calcification and cardiovascular risk

**Fig 1:**
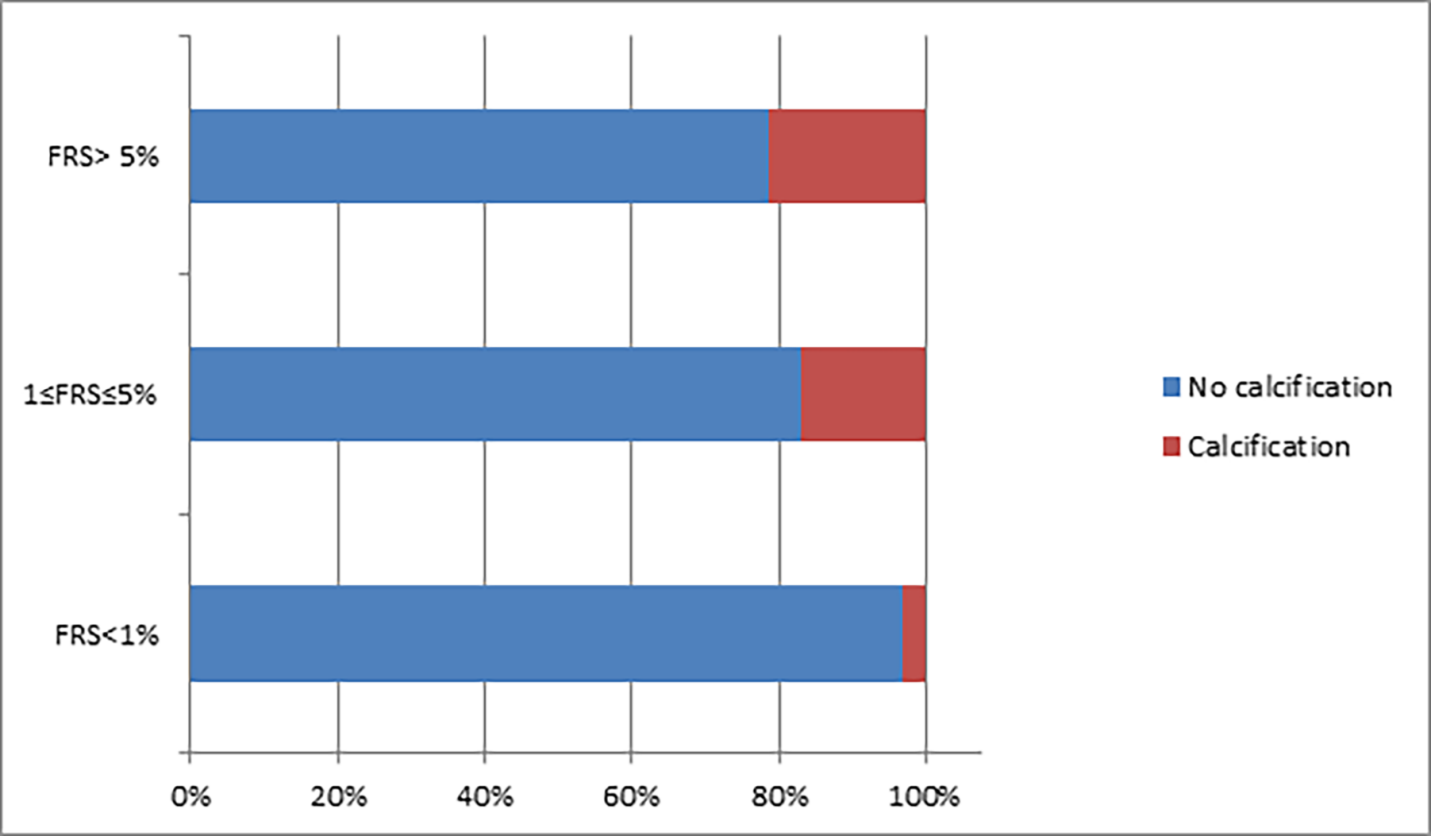
Calcification percentage by cardiovascular risk category. FRS = Framingham risk score.

Using our first approach for analysis, we found that FRS (categorized using three levels of severity) was significantly correlated with bi-categorical CACS (Cramer’s V = 0.24, p = 0.006), such that the proportion of participants presenting with CAC increased with the levels of FRS scores (Low—3%, Medium—17%, High—21%; p for linear trend = 0.01; Table 2 and Fig 1).

**Table 2:** Coronary calcification in a bi-categorical distribution by cardiovascular risk.

Cardiovascular Risk	No calcification	Calcification
**Low**	67 (97%)	2 (3%)
**Medium**	49 (83%)	10 (17%)
**High**	27 (79%)	7 (21%)

Table presents proportion of participants with coronary artery calcification according to levels of Framingham risk score. The difference in the proportion of CAC in the different groups was tested using Fisher’s exact test (p = 0.006), linear trend was tested using Mantel Haenszel’s test for linear trend. FRS = Framingham risk score; Low = FRS<1%; Medium = 1≤FRS≤5%`High = FRS>5%.

**Fig 2:**
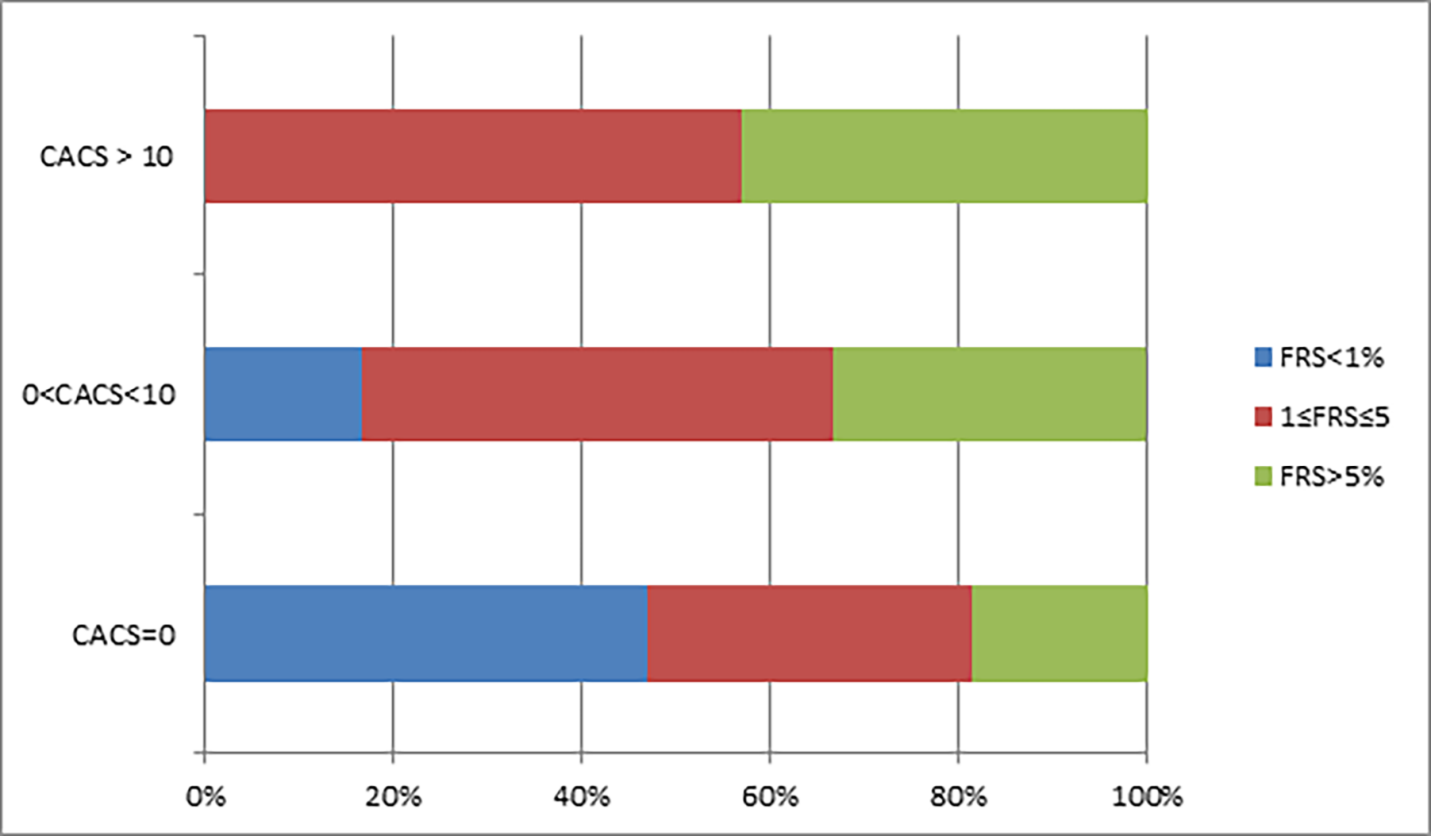
Categorical distribution of coronary calcification by categorical distribution of cardiovascular risk. Observing the proportion of the cardiovascular risk groups in the different calcification categories, it is evident that the more calcification demonstrated, the smaller the lowest cardiovascular risk group (colored in blue) becomes. p value for linear trend = 0.04. CACS = Coronary artery calcium score; FRS = Framingham risk score.

In our second approach, CACS and the FRS were separated each into 3 categories of increased score. The level of CACS was significantly correlated with FRS (Cramer's V = 0.18, p = 0.015) and prevalence of calcification increased linearly with FRS (Mantel-Haenszel’s test for linear trend p = 0.04, Table 3 and Fig 2).

**Table 3:** Coronary calcification divided to three categories by cardiovascular risk.

Cardiovascular Risk	CACS = 0	0<CACS≤10	CACS>10
**Low**	67 (97%)	2 (3%)	0 (0)
**Medium**	49 (83%)	6 (10%)	4(7%)
**High**	27 (79%)	4 (12%)	3(9%)

**Fig 3:**
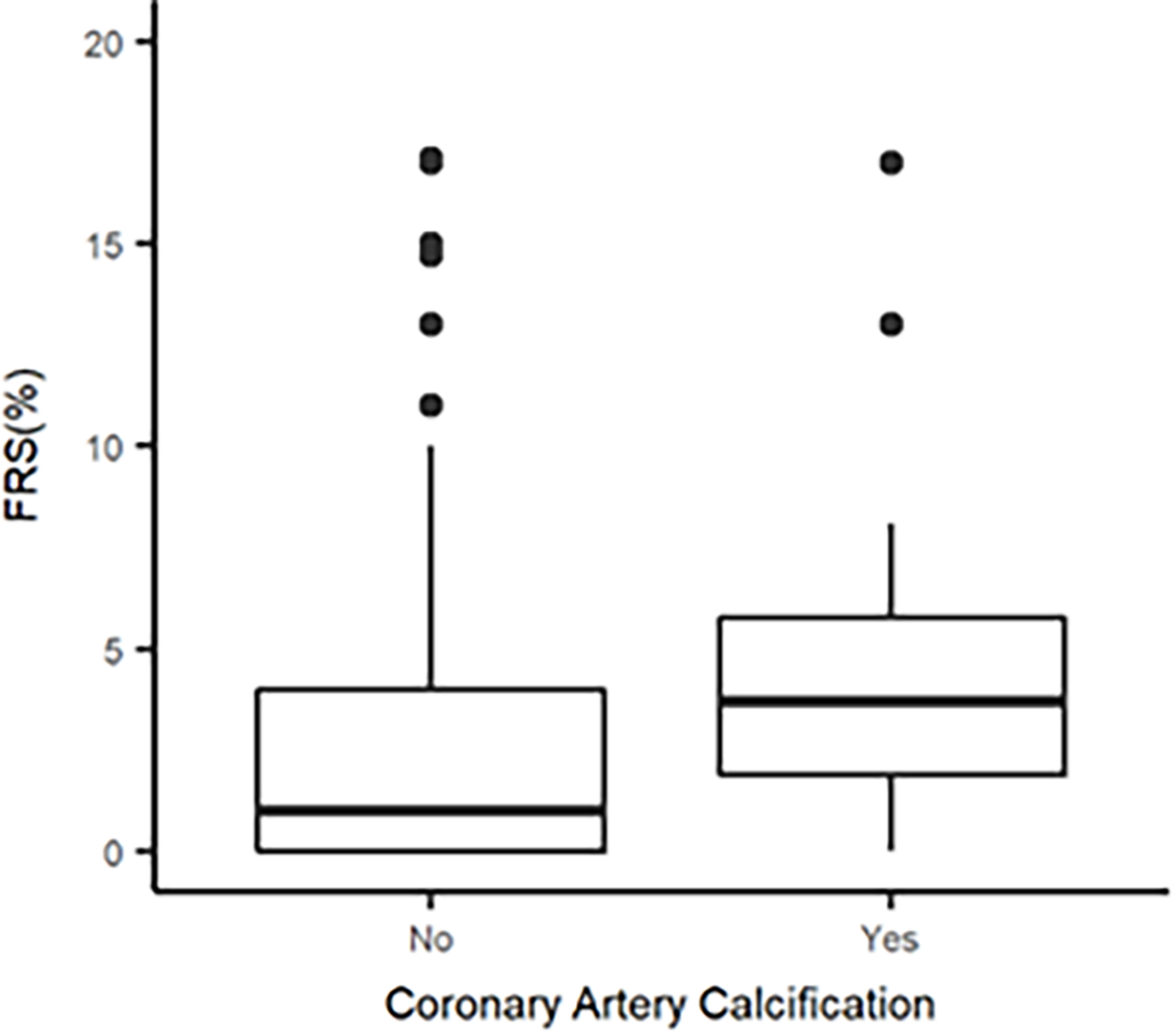
Calcification as a binary categorical variable by quantitative cardiovascular risk. Box plot showing bulk of higher Framingham risk score values in the calcification group. CACS = Coronary artery calcium score; FRS = Framingham risk score.

Finally, our third approach demonstrated that the presence of CACS, defined dichotomously, was significantly associated with the level of FRS, defined as a continuous measure (p = 0.002) (Fig 3).

### Coronary artery calcification and specific risk factors and measurements

In addition to the association between coronary calcification and cardiovascular risk, we investigated whether specific factors were more clearly associated with CACS score than others. This analysis found a significant positive correlation between increased age and smoking history and coronary calcification (p = 0.002 and p = 0.045, respectively). However, there was a noticeable trend for a correlation between male gender and BMI CACS. The results of these analyses are detailed in Table 4. Lastly, few participants in our study had type 2 DM (n = 6), or used antihypertensive (n = 5) or statins (n = 7). However, we did identify an association between statin use and risk for coronary calcification (p = 0.01 in Fisher’s test).

**Table 4:** Relation between specific factors and presence of coronary calcification.

	CACS>0	CACS = 0	P value
Age, mean (SD)	41.53 (6.65)	35.75 (9.5)	0.002ℓ
Sex (Females/Males)	3/16	54/89	0.074ϯ
History of smoking, n (%)	11 (57.9%)	48 (33.6%)	0.045*
Total cholesterol (mg/dL), mean (SD)	183.9 (39.33) [4.76 mmol/L (1.01)]	181.2 (36.98) [4.7 mmol/L (0.95)]	0.79ℓ
HDL (mg/dL), mean (SD)	42.8 (10.1) [1.1 mmol/L (0.26)]	45.2 (12.94) [1.17 mmol/L (0.33)]	0.37ℓ
Systolic blood pressure (mmHg), mean (SD)	126 (18.46)	124 (15.25)	0.69ℓ
BMI (Kg/m^2^), median (IQR)	29 (10.26)	24.4 (6.33)	0.09Ϟ
25-hydroxy vitamin D level (nmol/L) (normal range 20–100), median (IQR)	19 (14.5)	17 (12.875)	0.74Ϟ

HDL, High-density lipoprotein; BMI, Body mass index

* Chi-square test

ϯ Fisher’s exact test

ℓ T-test

Ϟ Mann-Whitney test

### Measurement biases

To rule out a possibility of measurement bias, 23 scans were randomly sampled by a chest imaging expert in order to test for inter-observer variability. We found 82.6% agreement between the two interpreters of this sample, the Cohen’s κ indicated good agreement between interpreters (κ = 0.636, p<0.001), and the Mcnemar-Bowker test did not detect a systematic difference between the interpreters (significance of 0.135). To rule out intra-observer variability, the first 30 scans analyzed were reanalyzed for a second time at the end of the study scan analysis, without any discrepancies between the early and the late interpretations.

## Discussion

In our study, we report a significant correlation between coronary calcification detected by non-gated MDCT and cardiovascular risk in young individuals. The association between coronary calcification, as quantified by the Agatston coronary artery calcium score, and cardiovascular risk, measured using the FRS, was investigated using three different approaches. All three approaches indicated CACS was significantly correlated with FRS.

Previously, the association between CAC in young individuals and early cardiovascular risk factors was demonstrated only using cardiac gated-MDCT [24]. This is the first report indicating CACS identified in non-gated MDCT is also associated with cardiovascular risk in a young population. These findings support the notion that the presence of coronary calcification in a random chest CT performed for non-cardiac indication in a young and healthy individual may have clinical and prognostic significance.

Young healthy individuals often undergo chest CT scans for non-cardiac indication–for example, following a trauma or during the investigation of a respiratory disease [26]. Sometimes these scans to reveal an incidental finding of coronary calcification, hitherto a finding of unknown significance which are not always reported by radiologists [22]. According to our study results, such findings likely have clinical importance, and may serve as a marker for cardiovascular risk status. This information can help inform proper medical management. A young and apparently healthy individual, without any prior cardiac investigations and with no previous cardiovascular risk evaluation, who presents with incidental coronary artery calcification in a chest CT scan, may benefit from early evaluation of CV risk factors and intervention, to help minimize the atherosclerotic process. As the FRS is associated with cardiovascular prognosis, our findings support the prognostic value of non-gated chest CT and its use as a screening test. Though our study indicates that CAC identified incidentally by non-gated CT is significantly correlated with a validated cardiovascular risk score, a longitudinal study would be required to demonstrate a conclusive link with clinical outcomes.

Our results also indicate that while some subjects with increased cardiovascular risk develop apparent CAC, others do not. Further research is therefore needed to further our understanding of the factors that contribute to the process of coronary calcification. In addition to known and established factors affecting the development of CAC, there are likely additional external and environmental factors, as well as endogenous factors, which may include genetic factors as well.

Our findings can help further our understanding of the factors that influence the process of coronary calcification process. Among known cardiovascular risk factors, we found that only history of smoking was independently and significantly associated with increased risk for coronary calcification. We had records for BMI values and serum vitamin D level only for some of the subjects (vitamin D values were collected only for 60 subjects and BMI values for 69). The association between vitamin D deficiency and high CACS values has been reported in studies performed in a population of diabetic patients [27, 28] and in the elderly [29]. Additionally, an association between high BMI values and coronary calcification in a young population has been previously reported, based on measurements obtained using both ECG-gated EBCT and MDCT [30, 31]. We were not able to reproduce similar results. We found a non-significant and small association between BMI values and coronary calcifications. Regarding both parameters, we should note the limited sample size. As well, we could not identify a significant correlation between hypertension and calcification as shown in prior studies [32, 33].

We found that statin treatment is significantly associated with calcification, although this finding should be viewed with caution as few included patients actually received statins. However, a recent post-hoc analysis of 8 prospective studies including 3495 patients (1545 treated with high-dose statin, 1726 with low-dose statin and 224 untreated) showed a significant association between statin use and increased coronary calcification despite atheromatous plaque regression as evaluated by coronary intravascular ultrasound [34]. Several limitations inherent to the retrospective design in a single medical center and to the limited sample size of our study necessitate cautious interpretation. Our results should be prospectively evaluated in a larger study before to be generalized.

## Conclusion

In conclusion, random chest MDCT in young asymptomatic individuals may detect CAC of clinical significance, and may help identifying patients who will benefit from early evaluation of cardiovascular risk factors.

## Supporting information

S1 Statistics Additional data for statistics. 19 patients with coronary calcifications.
(XLSX)

S2 Statistics Additional data for statistics. 143 patients without coronary calcifications.
(XLSX)
